# Sub-national assessment of aid effectiveness: A case study of post-conflict districts in Uganda

**DOI:** 10.1186/s12992-017-0251-7

**Published:** 2017-06-13

**Authors:** Freddie Ssengooba, Justine Namakula, Vincent Kawooya, Suzanne Fustukian

**Affiliations:** 1 School of Public Health, Makerere University, P.O BOX 7072, Mulago Hospital Complex, Mulago Hill Road, Kampala, Uganda; 2 Queen Margaret University, Institute for International Health and Development, Edinburgh, UK

**Keywords:** Aid-effectiveness, Aid assessment tool, Sub-national level, Post-conflict, Uganda

## Abstract

**Background:**

In post-conflict settings, many state and non-state actors interact at the sub-national levels in rebuilding health systems by providing funds, delivering vital interventions and building capacity of local governments to shoulder their roles. Aid relationships among actors at sub-national level represent a vital lever for health system development. This study was undertaken to assess the aid-effectiveness in post-conflict districts of northern Uganda.

**Method:**

This was a three district cross sectional study conducted from January to April 2013. A two stage snowball approach used to construct a relational-network for each district. Managers of organizations (ego) involved service delivery were interviewed and asked to list the external organizations (alters) that contribute to three key services. For each inter-organizational relationship (tie) a custom-made tool designed to reflect the aid-effectiveness in the Paris Declaration was used.

**Results:**

Three hundred eighty four relational ties between the organizations were generated from a total of 85 organizations interviewed. Satisfaction with aid relationships was mostly determined by 1) the extent ego was able to negotiate own priorities, 2) ego’s awareness of expected results, and 3) provision of feedback about ego’s performance. Respectively, the B coefficients were 16%, 38% and 19%. Disaggregated analysis show that satisfaction of fund-holders was also determined by addressing own priorities (30%), while provider satisfaction was mostly determined by awareness of expected results (66%) and feedback on performance (23%). All results were significant at *p*-value of 0.05. Overall, the regression models in these analyses accounted for 44% to 62% of the findings.

**Conclusion:**

Sub-national assessment of aid effectiveness is feasible with indicators adapted from the global parameters. These findings illustrate the focus on “results” domain and less on “ownership” and “resourcing” domains. The capacity and space for sub-national level authorities to negotiate local priorities requires more attention especially for health system development in post-conflict settings.

## Background

The aid-effectiveness discourse symbolized by the Paris and Accra declarations and the IHP+ aims to improve the governance of aid relations in developing nations. The Paris and Accra declarations on aid-effectiveness provided principles that essentially promote a well-functioning state with well-established national systems for planning, budgeting, priority setting and tracking and reporting development results [1]. This governance agenda is built on the assumption that the state has sovereign powers to coordinate local and international organizations. From this assumption, the aid-effectiveness discourse has focused on the national level capabilities to coordinate, track progress and build institutional mechanisms for governing aid relationships. Reality in many countries shows the limitation of this assumption and the focus at the national level. First, sub-national authorities such as local governments (Districts and Provinces) in decentralized nations provide a more instrumental and realistic settings for aid management [2, 3]. Many development actors like NGOs and bilateral funders relate directly with local governments to support development programs [4, 5]. As observed by Rowley et al. [6]; *“while the government calls for better coordination of efforts and the channeling of funds to the government (i.e., capacity building), donors and implementing agencies—especially those that are emergency-oriented—want to see the mortality rates go down as quickly as possible and believe that the best way to do that is through direct interventions.”(*[6] *page 20).*

However, local governments in post-conflict settings have more capacity gaps to effectively manage development actors – many of whom have superior powers from resources they control and legitimacy from influential international agencies and conventions [7].

Secondly, in post-conflict settings, many of these assumptions of state functionality and credibility are not realistic in the short to medium term. For example, in decentralized post-conflict settings, like Rwanda, Hayman [8] observes “*that central level achievements have been made but that “capacity for planning and implementation needs to be strengthened at local administration levels”* (Hayman [8] page 586). Depending on the causes and discourse of the social conflicts, state capability to coordinate the multitude of actors with rapidly changing interests and objectives is expected to be weak and contested [9]. The congested architecture of service providers, community development and fund-holding organizations in the context of weak state institutions to coordinate these is a powerful justified to revisit the operationalization of aid-effectiveness in the post-conflict settings especially at sub-national levels. Unfortunately little attention has shifted to the sub-national levels to improve aid effectiveness.

## Aid effectiveness in post-conflict settings

Development assistance and humanitarian aid remain prominent opportunities for functionalizing and rebuilding of health systems during and in the aftermath of social conflicts. This makes the agenda for aid-effectiveness in these settings a top priority for all organizations that are seeking to rebuild health and other social and development capabilities in post-conflict settings. Nonetheless, aid and its effectiveness in these settings present a bewildering array of complexities [10]. For example, Buse et al. [11] also indicates that the external aid many times fails to align with the local context and can undermine the health system [12]. Many authors reporting about the role of aid in post-conflict settings highlight the inevitability of missed opportunities and glaring ineffectiveness of resource use. Examples of proliferations of programs of limited duration, duplicative programming and sometime outright resource pilferage are common in the literature on post-conflict setting [13–16]. A multiplicity of organizations with fund-holding responsibilities and the relative autonomy of these agencies from state coordination are cardinal features that characterize post-conflict settings [17].

Global Health initiatives have in many ways innovated to by-pass the state level systems by dealing with non-governmental organizations and private sectors organizations in the aid dependent countries.

In response to the proliferation of international health financing architecture, the International Health Partnership (IHP+) was established to advance the aid-effectiveness agenda within the health sectors at national levels. IHP+ expects to mitigate the fragmentation of health governance systems of aid dependent countries [18]. The jury is still out on the success of the IHP+ in reigning in the global health initiatives to align to the national level coordination and governance for the health program.

## Aid governance in post-conflict northern Uganda

Government of Uganda policy on aid governance predates the Paris and Accra declarations. As noted by Jessica Ernst [19], the government of Uganda established partnership principles in 2001 with the aim of coordinating aid providers to the national poverty eradication action plans. In 2005, these efforts culminated in the Uganda Joint Assistance Strategy and institutionalization of SWAps in government sectors [19]. These processes did not provide specific guidance for post-conflict northern Uganda. Acholi sub-region started its post-conflict journey in 2006 after a 20-year civil war. The conflict was characterized by destruction of post-independence health system and other social infrastructure. During the conflict, the population was concentrated into protected camps where health services were provided by mostly non-state organizations with many expatriates and external resource support. At the peak of the conflict, some estimates show that there were over 300 health related organizations in Gulu district alone [20]. A more systematic programming for post-conflict reconstruction and developments of northern Uganda since 2007 has provided opportunity to finance development programs in the Acholi sub-region and to rebuild the functionality of sub-national governance systems of district/local authorities [21]. The financing has come from Uganda Government, and many international organizations like World Bank, bilateral donors from other governments and prominent non-state foundations and partnerships such as Gates Foundation and others. Given the decentralized governance structures in Uganda, and the prolonged period of conflict (1986 to 2006), the exercise of authority by the local governments in the post-conflict northern Uganda was identified as weak [22]. As a result of this, the Peace for Recovery and Development Plan (PRDP) of Uganda Government had an objective to re-establish state authority in the post-conflict region. For example, some of the programs of PRDP called for support to the local and district governance capacity to provide services and ensuring down and upward accountability ([23] page 32). Since 2006, when relative peace was achieved, many programs by government, NGOs and international organizations were set-up to promote the objectives of PRDP. For the period 2006 to 2013, over US 2.5 billions were reported as spend on PRDP objectives including health services in northern Uganda.

As a coordinating Center for PRDP, the Office of the Prime Minister (OPM) adopted the aid effectiveness principles enshrined in the Paris Declaration and Accra Agenda of Action to ensure more effective use of Government and aid funds to address PRDP objectives within the affected districts. Although aid effectiveness in post-conflict settings emphasizes the importance of state building – with emphasis given to rebuilding governance processes particularly to achieve basic service delivery, this has been limited to the national (state) level. A recent study evaluating the ‘applicability of the Paris Declaration in fragile and conflict-affected situations’ posted that aid effectiveness in fragile, transitional situations such as post-conflict in N Uganda will not be straight forward and needs to be “supplemented by a more fundamental concern with the effectiveness, accountability, responsiveness and legitimacy of the institutions of the state” ([22] page 4). We take this as a call for the development of appropriate tools to operationalize the assessment of Aid effectiveness at the sub-national levels – more so in contexts recovering from conflict – where classical plurality of aid actors bypass national states and interact with weak sub-national authorities to achieve humanitarian and development goals. In decentralized settings, the providers of aid to local governments have good reasons to bypass national level bureaucrats and directly work with local governments. Where decentralization is combined with post-conflict phenomena, local governments may not have all the capacity to coordinate or negotiate aid relationships.

In this study, the Local Governments in post-conflict northern Uganda are viewed as recipients of aid from Government of Uganda and foreign development agencies (donors) to implement social and development programs in their administrative areas (Districts). In Uganda, Districts have legitimate and constitutional mandate to ensure that services are provided to their communities ([22] page 4). Good district-level governance for service delivery (performance governance) is an expected outcome if the necessary relationships with other organizations (both public and private) are optimized to achieve strategic goals in the PRDP and in the National Health Sector and Investment Plan [24]. Health governance can be defined as the process of competently directing health systems resources, performance and stakeholder participation towards the goal of saving lives and doing so in ways that are transparent, accountable, equitable and responsive to the needs of the people (USAID [25]). Aid effectiveness agenda has established the minimum standards of governance relationship between aid recipients and providers of aid.

Five core principles form the basis for the Paris Declaration and Accra Agenda for governing aid relationships [26]. These principles have been developed following decades of experience of what works and what does not work to optimize aid effectiveness. Overall, these principles are aimed at improving the satisfaction and reporting among aid relationship. These principles have wide support across the development community. The five principles are captured in the following OECD definition of aid effectiveness:

*“It is now the norm for aid recipients to forge their own national development strategies with their parliaments and electorates (* ***ownership*** *); for donors to support these strategies (* ***alignment*** *) and work to streamline their efforts in-country (* ***harmonization*** *); for development policies to be directed to achieving clear goals and for progress towards these goals to be monitored (* ***results*** *); and for donors and recipients alike to be jointly responsible for achieving these goals (* ***mutual accountability*** *)”* [27]*.*

Although the above principles enjoy wide acceptance, their application in post-conflict situations present major challenges as the principals – central government, fund-holders and donors are more risk averse and unlikely to have a trusted local authority to provide credible leadership of the health system [28]. In these contexts non-state actors also work with relatively weak government counterpart to provide oversight and policy direction. To achieve some coordination, the non-state actors usually form their own coordination structures that might not be sustainable in the long term nor advance the ownership of the development programs by the communities.

## Study objectives and Methods

The general objective of this research was to explore the effectiveness of aid governance in the reconstruction of health systems in post-conflict sub-region in Northern Uganda. This work was undertaken as part of a broader set of studies trying to understand the processes of rebuilding health systems in post-conflict settings (https://rebuildconsortium.com) in Northern Uganda, Sierra Leon Cambodia and Zimbabwe. The specific objectives addressed in this paper area:

1. To develop a customized assessment tool for the status of aid-effectiveness that fits the sub-national level use in aid governance in post-conflict northern Uganda;
2. To assess the status of aid-effectiveness parameters within the network of organizations participating in health services delivery;
3. Identify the drivers of satisfaction (or dissatisfaction) with aid relationships among the major organizations that support service delivery in post-conflict Northern Uganda

The methods are organized around – 1) designing a customized instrument to assess the status of aid-effectiveness in the three post-conflict districts of Amuru, Gulu and Kitgum – in northern Uganda; 2) assessing inter-organizational aid relationships using the developed instrument; and 3) identify the drivers of perceived satisfaction with aid-effectiveness measures among different categories of agencies supporting service delivery in the study districts.

### Tools development

In this study, we developed a multi-item instrument to measure aid-effectiveness at the sub-national level. Customized measures were developed in the preparatory stage of the study (Table 2). Desk review of the literature was used to generate three to four items per domain of aid effectiveness. A stakeholder consultation workshop was used to refine and finalize the customization of the elements in the multi-element assessment tool. Stakeholders were identified from the district officials in the post-conflict northern Uganda, from Ministries of Health and Finance and from the Office of the Prime Minister (OPM). Others included local officials in the United Nations (WHO, UNFPA) agencies and from civil society agencies with activities in northern Uganda. At the workshop, the stakeholders were oriented about the aid-effectiveness agenda. In groups, the stakeholders were invited to discuss a prior list of questions proposed for the assessment of each principle of aid effectiveness at the district/subnational level. The groups were invited to refine, adjust or add to the proposed questions based on two criteria. First, groups were requested to ensure that each domain of aid-effectiveness is translated into relevant or equivalent sub-national concerns or concepts. For example, the “alignment” domain of Aid-effectiveness in OECD assessment is concerned with aid being in line with national priorities. The workshop groups were invited to consider the district-level issues or concerns regarding “alignment” of aid programs to district priorities. Secondly, groups were asked to propose new or refined set of questions to assess the district level status of each set of concern in the aid effectiveness framework. The number of items (questions) per domain was limited to two per domain to make the tool simple and convenient to the respondents. The main outcome of the workshop was a customized set of operationally relevant questions to use to assess the key aid governance domains at the sub-national level.

Table 1 shows the final questionnaire items for the tool that was generated from the consultation workshop. One summative question was added to the tool to assess the overall satisfaction, the respondent organization had for each external agency they listed as a partner for service delivery. For each questionnaire item, a standard 6-point Likert-type scale was develop ranging from 0 to 5 (“absence” to “highest”) to represent their opinion about each questionnaire item.

**Table 1:** Construct validity for the measurement instrument

AE Domain and definition	District level concerns	Questions developed to assess the domain at sub-national level
Ownership/Alignment • Developing countries set their own strategies for poverty reduction, improve their institutions and tackle corruption • Donor countries align behind these objectives and use local systems.	Respectful relationship that addressing local needs. Burden of diverse reporting requirements and delays in resource disbursements;	1. To what extent did resources received from XX address the main priorities of your organization? (PRIOR6) 2. To what extent was the organization able to negotiate with XX about the priority needs of your organization? (NEED6)
Harmonization Donor countries coordinate, simplify procedures and share information to avoid duplication.	Competition and duplication of activities; limited coverage, by-passing LGs in implementation	3. To what extent did XX organization use pre-existing admin procedures (e.g. reporting tools, bank accounts etc.) of your organization? (ADPROC6) 4. To what extent did XX coordinate with other organizations to support your organization? (COORD6)
Managing results Developing countries and donors shift focus to development results and results get measured.	Realistic targets and performance feedback	5. To what extent was your organization aware about the results expected by XX from your organization? (RESLTS6) 6. To what extent did XX provide feedback about the performance of your organization? (FEEDBK6)
Mutual accountability Donors and partners are accountable for development results.	Changing performance expectations; Delays in resource flow and disbursements	7. To what extent was the resources from XX come within the expected time last year? (EXPTIM6) 8. To what extent were the resources from XX based on a written agreement /contract/MOU with XX? (MOU6) 9. To what extent did your organization submit timely reports of activities to XX last year? (REPRTS6)
Overall evaluation Satisfaction with aid relationships	Satisfaction with dyadic aid relationships	10. Overall, to what extent are you satisfied about your relationship with XX organization? (RELATN6)

### Qualitative tools

The same likert-type questions were used to generate qualitative information. By asking the respondents to provide a reason or justification for the score they provided for each questionnaire item above, we generated the qualitative data to explain the basis of the respondents’ perception scores. Typically, the respondent was asked why he/she had selected a particular score on the scale. This question generated an explanation or justification for the score. Probes were used to get to the main reasons and context. Transcripts from the qualitative responses were read several times during analysis and recurring themes were identified within each domain of aid effectiveness (see Table 1). A coding framework was developed and shared by the study team members. The transcribed interviews were entered in ATLAS TI software and the text was coded using thematic coding. The themes were deductively arrived at from the domains of aid-effectiveness as assessed above and also inductively from each domain. For inductive analysis, we explored the reasons provided for high, medium and low scores. The explanations for these categories of scores were coded, sorted and organized into thematic categories arising from the data. The query reports were further scrutinized for patterns and emerging sub-themes. Quotations that epitomized the central themes were identified. Findings were then synthesized across the main themes, noting patterns and differences across the sub-themes.

### Survey of inter-organizational aid relationships

This was a 3-district case study using social network approach to data collection in post-conflict northern Uganda. The districts of Gulu, Kitgum and Amuru were purposively selected from the Acholi sub-region for the broader study where these objectives were nested [29]. The broader study sought to establish the relational architecture or networks among agencies supporting health service provision in the three districts. Data for this paper was collected using social network approach.

### Agency interviews

**Fig. 1:**
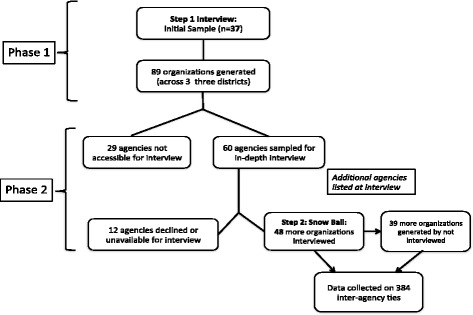
Sampling procedure for the 384 organization ties in the study

The findings are based on 85 organizations that were identified using a 2-stage snowball approach [27]. These two snowball stages (see Fig. 1) yielded 384 organization relationships (or “ties”). The unit of analysis in this study is the “exchange relationship” between any two organizations. For instance, an organization (ego) that received resources from three different external organizations (alters) was said to have three ties. Each tie was assessed separately during the interview with the tool (Table 1). During the second stage of interviews, many organizations listed as alters in the first stage were also visited and interviewed. Although some of the ties from first stage interviews were reciprocated (bidirectional), they were treated as new ties and assessed from the perspective of the respondent in that organization. This approach is recommended in the attempt to get the different experiences and perceptions that interacting pair of organization may have about each other.

The stage-1 interviews started with the District Health Office, and health provider organizations i.e. general hospitals and level III and IV health centers.

Both public and private providers were included. At this first stage, 37 organizations were interviewed. A list of 89 organizations were generated from stage-1 interviews. Of these, only 48 were available and consented to the stage 2 interviews – making a total of 85 respondents. Three exclusion criteria were used to prioritize the list of organizations for these stage-2 interviews.

The exclusions were 1) organizations that were assessed by respondents to have negligible contributions (below three on a scale of 1–10), 2) organizations not directly supporting the three selected services, and 3) organizations that were unreachable due to their location outside the study area. If not already listed from prior interviews, organization listed from stage-2 interviews were not followed up for interviews but information about them was collected and added to the dataset analyzed for this paper. Most of these third-order organizations were outside of the study area and many were located outside Uganda.

Our study started in 2013 - a period that can be characterized as the recovery or reconstruction phase in the post-conflict trajectory in Acholi sub-region [30, 31]. To limit the scope of the broader study, the survey of relationships was confined to three services only 1) HIV treatment and 2) maternal delivery services and 3) workforce strengthening. These services were selected on the basis of their prominence in post-conflict health system and the expected integration that is necessary to ensure optimal system effectiveness. The analysis pooled together data from all the 384 ties identified from 85 organizations that were interviewed.

A senior manager or a well informed official in the identified organizations was interviewed after securing informed consent. The interview followed a sociometric approach to data collection whereby some organization can be listed by more than one respondent and assesses separately by as many respondents [32]. For each inter-organization tie (relationship), the interview collected Likert-style scores based on the developed tool (Table 1) and qualitative data explaining the basis for each score.

## Results

The findings are organized into three sections. The first section provides the sample descriptions for the survey. The survey has two interrelated findings - 1) the validation and reliability of the instrument developed to assess aid effectiveness and 2) the survey findings regarding aid effectiveness relationships across 384 organizational ties. For the second objective, qualitative information is also provided to explain the partner of relationships by drawing on respondents’ experiences and examples.

### Sample description

**Fig. 2:**
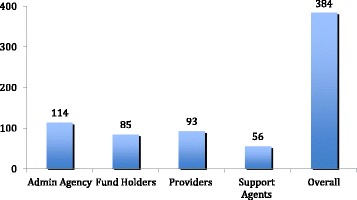
Functional categorization of organization ties in the study sample

The Fig. 2 below shows the sample and the main categories of the organizations in the three districts. Administrative agencies were mostly undertaking administrative and management functions in the district health system. These included District Health Offices, Administrative offices and non-governmental organizations that were set up to administer special health related programs and projects. Fund-holders organizations were mostly responsible for disbursing funds to other agencies in the district. Examples include recipients of grants from government and donors for health programs in the districts. Provider organizations were mostly hospitals and health centers concerned with health service provision (both public and private) in the districts. Support agencies were more mostly civil society organizations (CSOs) engaged in providing supportive services. The support functions mostly covered demand creation, logistics and capacity development. Although a few organizations had multiple roles, this categorization was guided by the most important function the respondent (ego) organization received from the relationships (tie) with the external organization (alter).

#### Construct validity of aid-effectiveness instrument

Inter-item correlations (Table 2) show high (above 0.32) coefficients and a high Cronbach’s alpha (0.862) suggests a high validity of the instrument.

**Table 2:** Correlations between items in the aid-effectiveness instrument

	1	2	3	4	5	6	7	8	9
1 PRIOR6^a^	1								
2 MOU6	0.59	1							
3 EXPTIM6	0.80	0.64	1						
4 NEEDS6	0.40	0.42	0.35	1					
5 RESLTS6	0.52	0.47	0.51	0.60	1				
6 ADPROC6	0.53	0.38	0.51	0.27	0.44	1			
7 FEEDBK6	0.37	0.39	0.39	0.39	0.50	0.31	1		
8 COORD6	0.32	0.22	0.24	0.30	0.31	0.25	0.37	1	
9 REPRTS6	0.41	0.36	0.37	0.41	0.45	0.39	0.45	0.27	1

^a^See bracketed variables names in Table 1 - right column

The table on item response (Table 3) below indicates that the change in questionnaire items does not create a worthwhile change in the reliability (alpha levels) if any item is deleted from the instrument.

**Table 3:** The item response reliability (*N* = 379): Cronbach’s Alpha = 0.862

	Mean	S.D.	Cronbach’s Alpha if Item is Deleted	Scale Mean if Item Deleted
PRIOR6^a^	2.57	1.834	0.837	19.37
MOU6	1.87	2.132	0.847	20.07
EXPTIM6	2.41	1.916	0.839	19.53
NEEDS6	2.75	1.777	0.853	19.19
RESLTS6	3.12	1.858	0.841	18.81
ADPROC6	1.65	1.819	0.853	20.29
FEEDBK6	2.62	1.862	0.852	19.31
COORD6	2.09	1.818	0.867	19.85
REPRTS6	2.86	1.89	0.854	19.08

^a^See bracketed variables names in Table 1 - right column

These findings provide confidence that the assessment instrument we developed to measure aid effectiveness at the sub-national level was reliable enough for the purpose. The content validity of the items as discussed in the methods section were also secured through stakeholder consultations.

**Fig. 3:**
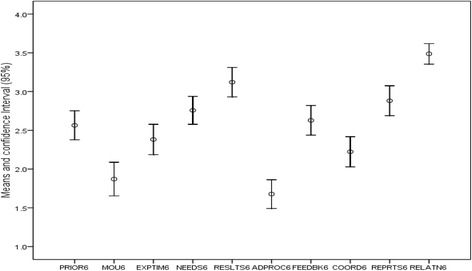
Mean and confidence interviews for items in the instrument (*N* = 373)

For item level exploration, Fig. 3 shows that the respondents had various experiences with regard to the aid-effectiveness parameters. High item-level means scores indicate that the status of aid-effectiveness was characterized by 1) address priority needs; 2) clarity of expected results; 3) providing feedback on performance; 4) providing timely performance reports; and 5) overall satisfaction. Low mean scores were related to 1) adherence to administrative procedures of the respondent organization and 2) presence of explicit contract for the relationship. These features broadly imply high attention to “managing results” and a low attention to “ownership/alignment”, “harmonization” and “mutual accountability” among inter-organization aid-relationships in the study districts.

#### Sub-national satisfaction with aid-effectiveness

To describe the pattern of aid relationships among the network of organizations in the three districts, a standard multiple regression analysis was carried out using “satisfaction with the aid relationship” as a dependent variable and the rest of the items in the instrument as predictor variables. The regression analysis was stratified by the major roles the alter organizations played in the district health systems – i.e. Administrative, Fund-holder, Service Provider and Support Agency. Figure 3 above provides the sample-wide means and confidence intervals for the items in the questionnaire. Descriptive statistics and correlations between the variables entered in the model are presented in Tables 2 and 4 below. A data driven description of aid-effectiveness was undertaken by running different regression models for each category of organization in the sample and an overall model that pools all the data together. The table below shows the four organizational categories and an overall model. The regressions results, overall and by organization categories, are presented in Table 4 below. The models show variations in the predictors of aid satisfaction across the category of organizations.

**Table 4:** Models: linear regression: dependent variable: satisfaction with alter

Models:	Admin Agents	Fund Holders	Health Providers	Support Agents	Overall
Coefficient	B	B	B	B	B
(Constant)	1.773	0.667	2.504	0.76	1.728
Priorities addressed (PRIOR6)	0.013	0.29***	−0.097	0.127	0.048
Having MOU (MOU6)	0.052	0.051	0.072	−0.122	0.024
Resource Timeliness (EXPTIM6)	0.05	−0.055	0.019	0.219*	0.009
Negotiate priorities (NEEDS6)	0.092	0.206***	−0.024	0.268**	0.105***
Expected results (RESULTS6)	0.24***	0.183**	0.307***	0.247*	0.253***
Different Admin procedures (ADPROC6)	0.004	0.055	−0.031	−0.061	−0.066*
Received Feedback (FEEDBK6)	0.005	0.066	0.108*	−0.151	0.113***
Coordinated support (COORD6)	0.105**	−0.107*	−0.034	0.17*	0.024
Submits reports (REPRT6)	0.052	0.186***	0.063	0.103	0.088***
R^2^	0.481	0.714	0.422	0.74	0.477
N	114	84	90	52	379
Model *P*-value	.001	.001	.001	.001	.001

* 5-10 percent *P* value, ** below 0.1 - 5 percent *P* value, *** below 0.1 percent *P* value

Table 4 shows that satisfaction with aid relationship was driven by:

1. The overall model indicates that “results” related variables provide the highest and significant predictors of aid satisfaction. Other factors being constant, awareness of the expected results in the aid relationship accounts for 25.3% increase in the perceived satisfaction in aid relationship. Receiving performance feedback and being able to negotiate priorities account for 11 and 10% respectively. Although marginally significant, the overall model shows that aid satisfaction is inversely related (6%) to the use of administrative procedures of the respondent organizations. By implication, there is aversion to the aid alignment theme in the aid-effectiveness’ agenda in the study districts.

2) The model focusing on organizations that perform administrative functions shows that the awareness of expected results and the extent external agencies coordinate (with others) to provide support to the respondent organization accounts for aid satisfaction of 24 and 10% respectively.

Qualitative findings indicated that the high scores in relation to ‘awareness of expected results’ was attributed to the central role that such agencies [administrators] play in negotiation for priorities in the district and in signing the explicit contracts. In most cases “expected results” are shared with administrators and are indicated clearly in the contractual agreements at this level.

*“I am aware to a very high extent because we agree; we set targets and really agree that ‘this should be the outcome’.* **[KII DHO Gulu].**

Satisfaction was reported to be low when administrators felt that they did not find flexibilities when negotiating their priorities with partners.

*“The partners, they come with their ‘jackets’ already fitted […]”* KII DHO Gulu.

Some administrators particularly those at sub national level implemented innovations such as ‘monthly health sector working group meetings’ to facilitate coordination of external support agencies. Such meetings were perceived to not only be platforms for learning about interventions of various organizations but also for coordination aid programs in the districts.

*“we have monthly health sector working groups where we meet with those other organizations to learn and support each other. KII Fund Holder Kampala.*

Although not indicated by the model, Qualitative findings also showed that administrators’ satisfaction is also predicted by report submission[receipt of reports] particularly those related to service delivery and financial accountability.

3) With regard to fund holders organizations, the model indicates that the main predictors of aid satisfaction are related to being able to negotiate aid priorities (20.6%) and to submit reports (18.6%) and being aware of expected results. Based on qualitative explanations, Fund holders perceived negotiation to be high when there was ‘flexibility’ with the district party they were negotiating with, when there was ‘agreement’, when they succeeded in securing administrative permissions to operate in the districts and when they had their priorities affirmed or adopted by the districts.

*“[…] because most of our interventions were aligned to the district development plan (DDP) and in most cases we could discuss with the DHO on what they are going to do, We had an agreement for the things that we could do that were in the DDP (district development plan) and … most of our things, fitted very well”* KII Fund Holder NGO Gulu.

On the contrary, aid satisfaction declines by 10.7% per unit increase in the coordination of external support agencies to the respondent organization all other factors in the model being constant. This was particularly true for those funding holder category.

*“…I think the coordination has been very poor… for all of them [* ***HIV related agencies]*** *because they all operate independently, they work independently. KII Admin. Agency National Level*

4) From the perspective of providers, the model indicates that the best predictor of satisfaction of aid relationship is being aware of expected results. This accounts for 30.7% of the variation in satisfaction in the aid relationship. Receiving performance feedback was also significant and accounted for 10.8% of the satisfaction in this sub-group. Providers preferred feedback to be instant, more regular and in some cases objective feedback so as to facilitate improvement in performance. Feedback in the form of appreciation was also preferred and perceived as a motivational factor for providers. Providers preferred feedback to be given to them directly other than indirectly, e.g. through the DHO’s Office because feedback provided through another entity rarely trickled down.

*“Feedback from [named CBO] is regular, because for the last 2 years they have held regular meetings on activities we have been doing. For example, in relation to ANC, we have monthly review meetings of performance. They tell us where our weaknesses are and where we have performed well […]”* KII Kitgum general hospital Kitgum.

*“As for Nu-health, feedback is very regular. It is monthly and quarterly …*” KII Health Provider Kitgum.

“*I would give them [Ministry of health] a [score of] five. They have verbally communicated about our good performance during support supervision, then they also gave us an award for being the third top most hospital in 2011 in Uganda, certified as third top most hospital in health service delivery in Uganda.”* KII Service Provider Kitgum.

However, satisfaction of providers was inversely related to being able to negotiate priorities. This is because negotiations with providers were reported to be rare and considered to be mostly undertaken by DHO’s office on behalf of the providers. By implications negotiations do not engender satisfaction. This may be due to power relationships, too little space available to negotiate already designed programs from upstream agencies and lack of direct contract relationship for the aid relationships at the sub-national level.

*“[…] Generally all these NGOs dealing with health services have to sit with the district and negotiate”* KII service provider Kitgum.

*“[…] The [named NGO] works according to the planned activities and the memorandum of understanding at the district level. Therefore, the priorities are planned and negotiated at the district level”* KII Service provider Amuru.

*“For all the DHOs I would give them a ‘four’ in terms of the negotiations but for the health centres, I give them zero[none] because the health centres do not take part in discussions with us unfortunately”* KII Support Agent Gulu.

The level of satisfaction of aid relationships with support agencies is predicted by three factors- negotiation of priorities, being aware of expected results and timeliness of resource receipts. These accounted for 26.8, 24.7 and 21.9% of the variation in the satisfaction in this subcategory. Coordination of support was also significant predictor of satisfaction (17%) for support agencies.

In addition to the eight items in the instrument, two more items – Satisfaction with relationships and extent of the respondent provides reports to the organization in question are included. Overall, the scores provided for satisfaction with aid-relationship are highest relative to all items in the instrument. The lowest scores were related to the use of explicitly contracts (or memorandum of understanding) and for the use of organization’s procedures (e.g. reporting templates, bank account) by external organizations. These indicate relatively poor alignment. The overall scale mean did not change much from 19.25 (out of 40).

Based on the qualitative explanations, the low scores for MOUs/ contracts/agreements could be explained by the fact that such arrangements were limited. For instance, they were reported to only exist between i) DHOs and fund holders [on behalf of all SPOs], ii) between Fund holders and Civil society organizations and iii] a few exceptions of SPOs that had been subcontracted by fund holders or CSOs. The DHO’s office signed explicit contracts on behalf of SPOs without the presence of any SPO representative. Explicit contracts between SPOs and DHO’s office were reported as non-existent and also rendered irrelevant as the latter is viewed as the supervisor of the SPOs or ‘being a part of them’.

*“We always have an MOU with [named hospital]. […] we were engaged with them, we [usually] sub-grant them to implement some programmes”* KII Support agent Kitgum.

*“[…] we don’t have any agreement or contact with the DHO’s office because they are our superiors”* [KII Service Provider Gulu.

Despite receiving the lowest scores, qualitative findings reveal that having explicit contracts such as MOUs was a very important aspect of the exchange relationship. This is because MOUs formalize the existence and operation of fund holders and CSOs within the district, stipulate activities that the agencies intend to implement for a given period of time, expectations that agencies have of the district as well as the district’s expectations of the agency. Additionally, the MOUs would also indicate the roles of different parties, the funding modalities and at times act as a negotiation platform and monitoring tool.

*“They don’t come to the district without signing any MOU and they follow strictly what they have signed. That is to say, the period that they are working and all that”* Admin. Agent Kitgum.

*“[…] because you cannot get all that money without a written document so there is a proposal between donor XX, YY and ZZ”* KII Fund Holder (Kampala).

*“Well, we had a contract, an MOU with the district saying that we would provide technical assistance and we had also some obligations in terms of using their human resource and established infrastructure and they could avail us time for our technical staff to work[…]”* KII Fund Holder Gulu.

## Discussion

As more aid agencies target non-state actors and sub-national administrative authorities especially in post-conflict settings, efforts to assess and track aid-effectiveness need to move along the same path. Downstream operationalization of aid effectiveness agenda is vital in the realization the objectives of the International Health partnership (IHP+) especially in decentralized settings. IHP+ expects to mitigate the fragmentation of health governance systems especially in aid-dependent countries [18]. Like other post-conflict health systems, the northern Uganda, − the study area display-marked congestion of aid and development workers within a context of weak public administration systems [33]. The era of sustainable development goals (SDGs) calls for distributed governance – where all duty-bearers (at national and sub-national level) need to effectively participate in the governance of their own scope of influence. Tools that support governance at sub-national levels contribute in ensuring that the distributed governance agenda is realized in optimizing the effectiveness of aid programs [34].

The findings in this study demonstrate the performance of a custom-made instrument developed to assess the effectiveness of aid relationships at the sub-national level. The engagement of national and district-level experts close to the aid industry in the health sector was able to support the development and customization of the global tools for aid-effectiveness to sub-national ones. Key to this customization was the operationalization of the global concerns to their equivalents at the sub-national level (Table 1). Overall, finding show that the aid-effectiveness concepts as developed in the Paris Declaration can be customized and used to assess the aid-relationships among the network of organizations involved in service delivery at the district level. The custom tool used in our study shows a high level of reliability with a Cronbach’s Alpha = score of 0.862. As discussed by Mutale et al. [35] and Shorten et al. [18] rigorously monitoring of aid practices is complex at the global level and they called for building capacity in-country to strengthen aid monitoring. The tool presented here is aimed at advancing the capacity to track the development of aid-effectiveness and IHP+ at sub-national levels [35].

Using the developed tool, the status of aid-effectiveness in the three post-conflict districts provides a pattern that the “result agenda” is prominent in the aid-relationship. High scores (above 3) were found in relation to awareness of results, reporting performance. In addition, there was high overall satisfaction with aid-relationships in these districts. Low scores were related to presence of contracts or MOU, use of or aligning to procedures of recipient organization and coordination of agencies in these districts. These results confirm an unbalanced implementation of aid-effectiveness agenda. More emphasis of aid results is well documented in the literature especially in the advent of the MDGs [36, 37]. This study shows that processes that make the delivery of aid more efficient remain weak or less optimized in the study distracts. For example, coordination to avoid duplication, use of existing procedures to optimized harmonization and alignment, and ensuring that there are explicit contractual obligations to support mutual accountability are least invested. Other findings reflect the role of power in the aid relationships – posting that interests of the powerful groups in aid-relationships are optimized while the interests of the recipients remain less optimized [38]. Moderate scores were observed for the ability to negotiate local needs, timely resource provision, and provision of performance feedback to service networks in our study districts. These are areas that are doing unspectacularly well but need more investment at the sub-national levels – especially in the post-conflict health systems where this study was carried out.

The findings in this paper were validated by sharing them with the district network of actors from whom the study was done. The dialogues at these meetings generated insights into how to negotiate for the priorities of district by those in authority and how to communicate the expectations of funding agencies to the rest of the network – especially in situations without formal contracts or memoranda of understanding between providers of aid and service providers. The most intractable issue discussed in these meeting was the high prevalence of delayed financial remittances from fund-holders – both government and non-governmental agencies. Close links between the money provided, the results expected and time within which to implement activities was a recurrent theme in the dialogue meetings. Overall, this implied that the timing of financial disbursement was vital in ensuring aid effectiveness – as is the magnitude of the funds disbursed. It also emerged that the fund-holders did not have full information about when they would get the funds from their principal sources –government, bilateral donors and through global health initiatives (GHIs) like The Global Fund, Global Alliance for Vaccines Initiative (GAVI) and other GHIs agencies. The uncertainty of funding upstream was multi-layered – thus making the district-level networks unable to find effective solutions at their level. Similar findings have been found in Kenya among implementers of donor-financed programs [39].

Engagements at the national and district level indicated that the tool developed here was complimentary to an already existing tool. The district league table is well established [40] as a tool to rank the performance districts using mostly service output data. The aid assessment tool developed here provides an essential ingredient to understand the performance of districts from the perspective of the “aid relationships” in the network of organizations that are implementing public programs. Overall, district and national level health managers optimistically received the tool as a way to monitor aid relationships and to provide the local authorities with a basis for articulating their governance and interactions with providers and users of health related aid/grants – both from government and non-government entities.

## Conclusion

As the health system in northern Uganda rebuilds after conflict, it is crucial that local authorities develop capacity to orchestrate the multiple actors involved in post-conflict programming and those involved in the provision of health services. Tools like one developed and locally validated in this study can contribute to generating essential data upon which to build stakeholder dialogue to advance aid effectiveness at the sub-national levels. The study demonstrates the value of operationalizing the aid-effectiveness discourse at sub-national levels where most aid actors operate in decentralized settings like Uganda.

## Abbreviations

DHO: District Health Office(r)
CSO: Community service organization
SPOs: Service provider organizations
MOU: Memorandum of understanding (or contracts)
KII: Key informant interviews
PRDP: Peace, Recovery and Development Plan
